# Intersystem Crossing of 2-Methlypyrazine Studied by Femtosecond Photoelectron Imaging

**DOI:** 10.3390/molecules27196245

**Published:** 2022-09-22

**Authors:** Naipisai Wumaierjiang, Bumaliya Abulimiti, Fengzi Ling, Mei Xiang, Xulan Deng, Jie Wei, Bing Zhang

**Affiliations:** 1 Xinjiang Key Laboratory for Luminescence Minerals and Optical Functional Materials, School of Physics and Electronic Engineering, Xinjiang Normal University, Urumqi 830054, China; 2 State Key Laboratory of Magnetic Resonance and Atomic and Molecular Physics, National Center for Magnetic Resonance in Wuhan, Innovation Academy for Precision Measurement Science and Technology, Chinese Academy of Sciences, Wuhan 430071, China

**Keywords:** methylpyrazine, photoelectron imaging, intersystem crossing, pump–probe, time-resolved spectroscopy

## Abstract

2-methylpyrazine was excited to the high vibrational dynamics of the S_1_ state with 260 nm femtosecond laser light, and the evolution of the excited state was probed with 400 nm light. Because it was unstable, the S_1_ state decayed via intersystem crossing to the triplet state T_1_, and it may have decayed to the ground state S_0_ via internal conversion. S_1_-to-T_1_ intersystem crossing was observed by combining time-resolved mass spectrometry and time-resolved photoelectron spectroscopy. The crossover time scale was 23 ps. Rydberg states were identified, and the photoelectron spectral and angular distributions indicated accidental resonances of the S_1_ and T_1_ states with the 3s and 3p Rydberg states, respectively, during ionization.

## 1. Introduction

Molecules are excited to electronic excited states after absorbing ultraviolet light, and the excited state can rapidly initiate ultrafast non-adiabatic process with an energy flow, which can lead to some excited-state photochemical processes that are harmful to living organisms [1,2]. Intersystem crossing and internal conversion are the primary excited-state relaxation pathways essential for many biological processes, such as photosynthesis and DNA self-repair. Hence, it is important to study electron relaxation dynamics in these non-adiabatic processes [3,4]. Because molecules can be ionized from arbitrary excited states with various multiplicities, there is a need to observe the levels of dark states in real time via intersystem crossing (ISC) [5]. Quantum-yield and fluorescence measurements can reveal non-adiabatic dynamical information. However, there is an urgent need for a comprehensive method to elucidate all of the dynamics.

Pyrazine aromatic hydrocarbon compounds are stable, highly toxic, carcinogenic, teratogenic, and widely distributed in nature [6]. They are important in biological research, pharmaceutical and pesticide production, chemical synthesis, and environmental protection. They can be electronically excited with ultraviolet light, and the excited state can rapidly initiate ultrafast non-adiabatic process with an energy flow that is important in natural photophysical and photochemical processes. Related theoretical and experimental studies have focused on aromatic hydrocarbon compounds such as benzene and its derivatives. With the development of laser technology to explore chemical reactions on the femtosecond timescale, a new, extremely fast reaction channel has been observed in benzene. Radloff [7] et al. studied the internal conversion of its excited S_2_ state and observed that it decayed to the S_1_ state in 40 ± 10 fs. The vibrationally excited S_1_ state was then internally converted to the S_0_ state in 6.7 ± 0.3 ps. Fielding [8] et al. studied the channel-three effect of the S_1_ state of benzene and found that when it was excited to a level 3000 cm^−1^ above the S_1_ state, approximately 20% of the excited-state population was transferred to the T_1_ state. At the same time, approximately 6% of the population oscillated back and forth between the S_1_ and T_2_ states. They estimated that these processes all occurred within a few-hundred femtoseconds. After being excited to the S_2_ state, benzene is rapidly converted to the S_1_ state. The non-adiabatic transitions of S_1_ to the S_0_ or T_1_ states generally occurs in nanoseconds, and the internal conversion and intersystem crossing compete with each other [9]. Many chemical reactions occur in the femtosecond timescale [10,11,12,13,14,15]. The corresponding lifetimes of the S_2_ and S_1_ states are usually within 100 fs and between 4.3 and 8.8 ps, respectively, and there is a “channel-three” effect that plays a key role. (Because there is no standard kinetic process associated with it, Callomon [16] et al. referred to this unknown process as “channel three”.) This effect refers to the region 2000 cm^−1^ above the excited state. The lifetime in this region is much shorter than that of the S_1_ state band. After molecules are excited to this region, the lifetime suddenly becomes much shorter because of intra-transitions or inter-system crossover; hence, the channel-three effect [17].

The excited-state dynamics of nitrogen-containing aromatic hydrocarbon compounds, especially pyrazine, have attracted great attention [18,19,20] with regard to their ultrafast and radiation-free dynamics [21]. However, research on the dynamics of electronically excited 2-methylpyrazine has been less frequent. Here, because the dynamics in the channel-three region in 2-methylpyrazine is still controversial [22,23,24], we performed time-resolved photoelectron imaging. The non-degeneracy of 2-methylpyrazine should lead to less symmetry prohibition and a higher vibrational level density. Therefore, we expected to observe non-adiabatic kinetics, and we investigated the channel-three effect of the S_1_ state using femtosecond pump–probe photoelectron imaging. The Rydberg states were identified via photoelectron spectroscopy and angular distributions.

## 2. Materials and Methods

Experiments were performed with a home-made optoelectronic imaging system consisting of an ultrasonic molecular beam sampling system in a vacuum chamber, an ion lens, a timing controller, two-dimensional image detection, and a signal-acquisition and data-processing system [25]. The vacuum system had a beam-source chamber and an ionization chamber evacuated by a molecular pump (F200/1200) with a rotational speed of 400 rpm and a pumping speed of 1200 L/s. A high vacuum of 10^−6^ Pa was maintained when there was no sample injection. When the pulse valve was opened to inject the sample, the vacuum in the beam-source chamber was 1.0–3.0 × 10^−4^ Pa, and that of the ionization chamber was 10^−5^ Pa or 10^−6^ Pa. To protect the electrons from external electromagnetic fields, the ionization chamber was shielded with a μ-metal (iron-nickel alloy) layer.

Seed light generated by a self-mode-locked Ti:sapphire oscillator was amplified with a chirped-pulse regenerative amplifier that had an 800 nm fundamental frequency output at 1 kHz, a pulse width of 100 fs, and single-pulse energy of 4.5 mJ/pulse. The fundamental was divided into two beams, one of which was used to pump a traveling-wave optical parametric amplifier that generated a 260 nm pump pulse for the resonant excitation of the high vibrational dynamics of the S_1_ state band of 2-methylpyrazine (30,944 cm^−1^). The other fundamental beam was frequency-doubled to 400 nm for use as a probe pulse. The polarizations of the pump and probe beams were adjusted parallel to the detector plane via a variable-wave plate and a half-wave plate, respectively. A series of photoelectron binary images were collected at various pump–probe time delays, and three-dimensional (3D) images were reconstructed with the BASEX transform [26]. In the experiment, 260 nm pump light and 400 nm probe light were used. The 2-methylpyrazine molecule first absorbs a pump light to excite it to an excited state, and then absorbs two probe lights to generate ionization. The cross-correlation function of the 260 nm pump and the 400 nm probe pulses was determined to be 260 fs.

In the experiment, 99.9% 2-methylpyrazine was used without further purification. Helium at a background pressure of 2 atm was used as the carrier gas for the saturated vapor of the liquid sample. The mixed gas was sprayed into the beam-source chamber through the pulse valve to form an ultrasonic molecular beam that was collimated by a skimmer before entering the ionization chamber. It interacted there with the laser beams between the repelling and accelerating poles of the ion lens. The 2-methylpyrazine was ionized by the pump and the probe pulses to generate photoelectrons and photo-ions. By selecting the appropriate voltage polarity and voltage ratio, the ion lens would accelerate and focus the generated photoelectrons and photo-ions onto a two-dimensional, position-sensitive detector composed of two microchannel plates and a fast-response P47 phosphor screen to form high-resolution photoelectron or ion images. The images were collected with a CCD camera mounted to the back of the detector. A photomultiplier tube was also used to collect the photoelectron and ion mass spectrometry signals. The original two-dimensional image was reconstructed via BASEX transformation to a 3D distribution. The entire experimental sequence was controlled by a DG535 controller.

## 3. Results and Discussion

Since 2-methylpyrazine only reported the experimental value of the energy of the S_1_ state, and no experimental and theoretical values were found for other excited states, density functional theory (DFT) was used with a B3PW91/6-311++G (d, p) basis set. Transition characteristics, vertical excitation energies (Eexc), and corresponding oscillator strengths (f) of various electronically excited states were calculated, as summarized in Table 1. It is found that the oscillator strength of the S_2_ state is very small, so it is believed that our pump light excites the high vibrational state of the S_1_ state.

Experimentally, double-light and single-light mass spectrometries were performed. The pump and probe pulse energies were controlled so that there was almost no signal for a single light. At zero-time, the double-light mass spectrum had two peaks, and the stronger signal corresponded to the parent (C_5_H_6_N_2_^+^) ion. There were also weaker signals corresponding to ionized fragments of the parent ions. The calculated signal intensity of the parent ion was several factors of ten higher than that of the fragment ions, indicating that photoelectrons from the fragment ions were negligible. In addition, the intensities of the parent and fragment ion signals changed similarly with time. Therefore, most of the photoelectron signals were directly ionized from the parent ions. 

**Figure 1:**
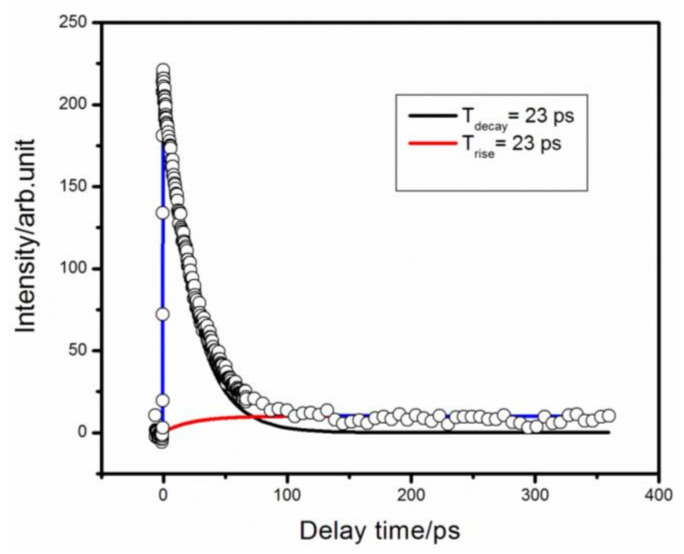
Time decay of the parent ion obtained with 260 nm pump and 400 nm probe detection. The circles are experimental results, and the solid lines are fits to a convolution of a Gaussian cross-correlation function and exponentially decaying and exponentially rising functions. This resulted in different time constants.

Figure 1 is a parent (C_5_H_6_N_2_^+^) ion signal as a function of time. It was fitted via a convolution of an exponentially rising and exponentially decaying functions and a Gaussian function. The convolution was necessary because in the femtosecond time-resolved experiment, both the pump and probe light pulses had Gaussian pulse widths, while the photoelectric conversion devices had certain response times. Thus, the recorded ion signal intensity was a convolution of the real signal of the excited-state layout number over time with the correlation function of the pump and probe light pulses. For example, a molecule could be excited to state A. However, because A is unstable, it will decay over time. That is, the A state will decay to another excited state, state B, and state B may also decay to the state C over time. The rate constant of the A-state decay can be denoted as g_1_, and that of the B state to the C state can be denoted as g_2_. Hence, an equation will have the form:

(1) $$\frac{dA(t)}{dt}=-g_{1}A(t)\Rightarrow A\left(t\right)=A\left(t_{0}\right)e^{-g_{1}t}$$

(2) $$\frac{dB(t)}{dt}=g_{1}A(t)-g_{2}B(t)\Rightarrow$$

(3) $$B\left(t\right)=\frac{g_{1}A(t_{0})}{g_{1}{-g}_{2}}\left(e^{{-g}_{2}t}\right.-\left.e^{{-g}_{1}t}\right)$$

(4) $$g_{2}=0\Rightarrow B\left(t\right)=A\left(t_{0}\right)\left(1-\left.e^{-g_{1}^{}t_{}}\right)\right.$$

(5) $$\frac{dC\left(t\right)}{dt}=-g_{2}B\left(t\right)\Rightarrow C\left(t\right)=\frac{g_{1}g_{2}}{g_{1}-g_{2}}A\left(t_{0}\right)\times \left[\frac{1}{g_{2}}\right.\left(1-\left.e^{-g_{2}t}\right)\right.-\left.\frac{1}{g_{1}}\left(1-\left.e^{-g_{1}t}\right)\right.\right]$$

In the above equations, the variation in the number of layouts of excited states with time is a single-exponential decay and a single-exponential rise function. Because the femtosecond pump and probe pulses were Gaussian, the correlation function of the two Gaussian pulses was still Gaussian. In the experiment, the correlation function of the pump and the probe pulses was measured first, and then the mass-spectral signals under the measured pump and probe time delays were fitted by the convolution of the exponential and Gaussian functions to obtain the precise lifetime of the molecule in the excited state:

(6) $$I_{sig}=\sum_{i}A_{i}\exp(-t/\tau_{i})⊗\frac{1}{\sigma \sqrt{2\pi }}\exp\left[-\frac{{\left(t-t_{0}\right)}^{2}}{2\sigma^{2}}\right]$$

In Equation (6), $A_{i}$ is the amplitude, $\tau_{i}$ is the lifetime, σ is the full-width at the half-maximum of the correlation function, and $t_{0}$ is the zero-point time that represents the convolution operation.

As shown in Figure 1, the time-resolved ion signal could be fitted by a convolution of an exponential rise, an exponential decay, and a Gaussian function to obtain an exponential rise and decay time of 23 ps. The 260 nm pump light excited the high vibrational dynamics of the S_1_ state of 2-methylpyrazine. In pyrazine, the S_2_ state is short-lived at less than 20 fs. We acquired the absorption spectrum of 2-methylpyrazine, which showed broadband absorption near the S_2_ state. Therefore, we deduced that the S_2_ state of 2-methylpyrazine was also short-lived. However, we did not observe this transient state in experiments. The pump–probe cross-correlation function was approximately 200 fs, which may have limited the time resolution of the short S_2_ state lifetime. We considered the lifetime of the S_1_ state after the transition from the excited S_2_ state. The ion signal could be fitted by the convolution noted above with a decay of 23 ps, which was attributed to the decay of the S_1_ state. A 23-ps rising signal was also observed with the decay of the S_1_ state, which probably reflected the layout of the T_1_ state. To better understand the decay process following the 260 nm pump pulse, we collected photoelectron images for different pump–probe time delays.

**Figure 2:**
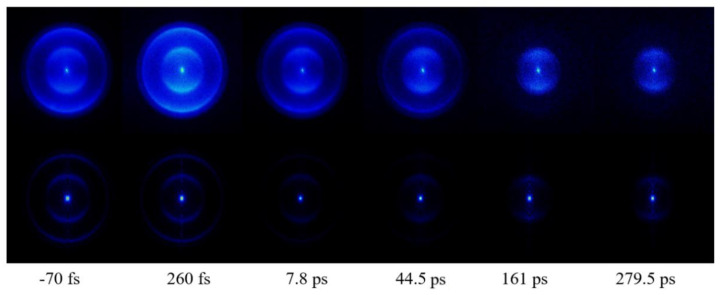
Time-resolved raw photoelectron images (**upper row**) and BASEX-inverted images (**lower row**) at various time delays between the 260 nm pump and 400 nm probe pulses.

Figure 2 shows photoelectron images obtained for various pump–probe time delays. The upper row shows original images, which are projections of the 3D photoelectron distributions on the two-dimensional detector. The lower row shows the corresponding reconstructed 3D images after BASEX transformations. These images consisted of four rings, where the outer three rings disappeared at longer pump–probe time delays, while the innermost ring remained. To examine the decay processes more clearly, photoelectron spectra at different time delays were obtained from the photoelectron images.

**Figure 3:**
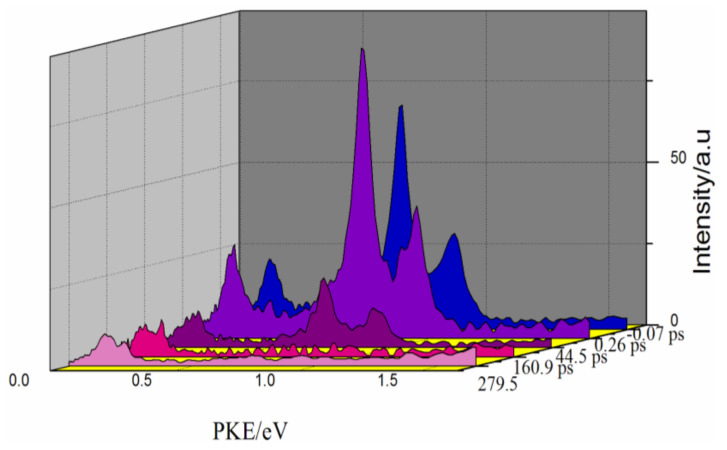
Photoelectron kinetic energy distributions (PKEs) at various time delays.

**Figure 4:**
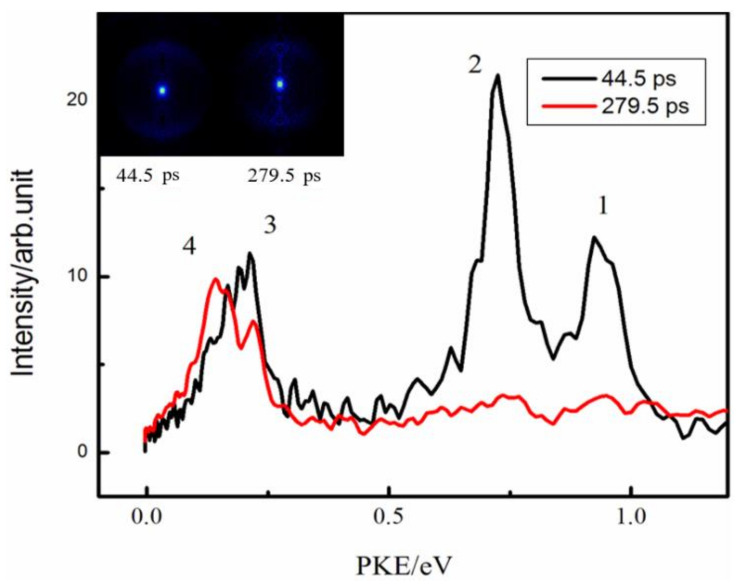
Two-dimensional photoelectron images at Δt = 44.5 ps and Δt = 279.5 ps. The left half shows the original image, and the right half is the BASEX-transformed image. The photoelectron kinetic energy distributions were extracted from the images. When pumping at 260 nm, the first, second, and third peaks come from the ionization of the S_1_ state, and the fourth peak comes from the ionization of the T_1_ state.

We obtained photoelectron spectra at different time delays from the photoelectron images, as shown in Figure 3. There were four peaks: the first three peaks decayed over time, while the fourth peak near 0 remained over time. The photoelectron images and corresponding photoelectron spectra at 44.5 ps and 279.5 ps are shown in Figure 4. From the spectra, there were three photoelectron peaks at 44.5 ps. All three decayed over time, while the fourth peak appeared at 279.5 ps. Because the S_1_ state had already decayed after 279.5 ps, the fourth peak was attributed to the ionization of the T_1_ state. The S_1_ state decayed at the same time as the T_1_ layout. When pumping at 260 nm, intersystem crossing was still the main channel for S_1_ decay. The T_1_ lifetime in 2-methylpyrazine was likely to be similar to that in pyrazine measured in the time-resolved photoelectron imaging experiments of Wang et al. [27]. We observed that the S_1_ state decays to the T_1_ state through the intersystem crossing process after being laid out. After S_1_ is laid out, it is also possible to decay to the high vibrational state of the S_0_ state through the internal conversion process. However, the high vibrational state of the S_0_ state has a vibrational energy of 4.76 eV, and the 400 nm probe light cannot ionize such a high vibrational state of the ground state. Therefore, in our experiment, there is no way to observe the channel that converts S_1_ to S_0_, but we believe that the internal conversion from S_1_ to S_0_ after the S_1_ layout is also an important channel.

In the photoelectron kinetic energy distributions in Figure 3, all four peaks were sharp. Hence, the excited state of 2-methylpyrazine had an accidental resonance with a Rydberg state during ionization. The 2-methylpyrazine molecule absorbs probe photons to resonate with the Rydberg state. For example, the presence of the 3s and 3p Rydberg states observed in pyrazine and pyridazine enhanced (1 + 2’) resonant multiphoton ionization, and the angular distribution anisotropy of the photoelectrons generated from the 3s Rydberg state was stronger than that of the 3p Rydberg state. Therefore, we expected to observe similar behavior here. Ionization from the Rydberg state to the cationic state involved Δv = 0, and the photoelectron kinetic energy (PKE) could be expressed as [28]:

(7) $$PKE=T_{R}+\hbar \omega_{2}-IP=\hbar \omega_{2}-\frac{R}{{\left(n-\delta \right)}^{2}}$$

where *IP* is the ionization potential, *hω*_1_ and *hω*_2,_ are the pump and probe photon energies, respectively, *T_R_* is the Rydberg state vibrational energy, *n* is the principal quantum number, *δ* is the quantum defect, and *R* is the Rydberg state constant (13.606 eV).

The four peaks in Figure 4 are located at 0.95 eV, 0.72 eV, 0.22 eV, and 0.14 eV. From Equation (7), the quantum defects of the four Rydberg states are 0.48, 0.61, 0.83, and 0.87, respectively. Because the value of the quantum defect δ that generally corresponds to the s orbital of the Rydberg state is approximately 0.9–1.2, that for the p orbital is between 0.3 and 0.5, and that for the d orbital is 0. The value of the quantum defect also indicated that the fourth peak was likely to arise from an accidental resonance with the Rydberg state. Table 2 lists the Rydberg state assignments and δ values of pyrazine and 2-methylpyrazine. By comparing these Rydberg state assignments for pyrazine and 2-methylpyrazine, the first and second peaks came from the 3p Rydberg state, the third peak was from the singlet (S_1_) 3s Rydberg state, and the fourth peak was from the triplet (T_1_) 3s Rydberg state.

**Figure 5:**
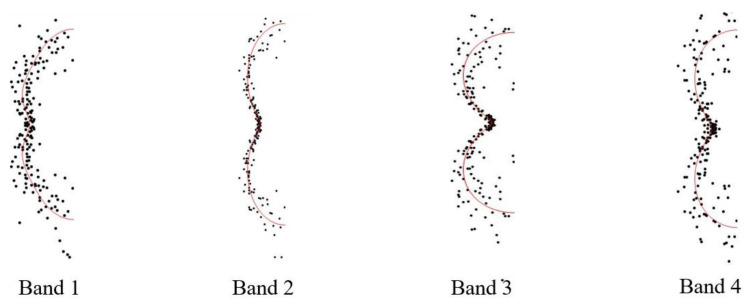
Photoelectron spectral and corresponding angular distributions.

Various photoelectron angular distributions reflect different molecular-orbital characteristics. Figure 5 shows that the angular distributions of the different rings come from different intermediate states. In the atomic images, pure p-waves are generated from s-state ionization, and p-state ionization generates s- and d-waves. The interference of s- and d-waves will weaken the angular distribution anisotropy of the photoelectrons. Hence, in general, the photoelectron angular distribution anisotropy from the s state was stronger than that of the p state. The same holds true for Rydberg states in molecules with similar electronic configurations. Therefore, the photoelectron angular distributions were also a good means to understand the Rydberg states.

When exciting the high vibrational dynamics of the S_1_ state, we still observed intersystem crossover. After the S_1_ state was laid out, its attenuation to the triplet T_1_ state was still a very important channel. To study the competition between S_1_-to-T_1_ intersystem crossing and internal conversion from S_1_ to S_0_, we used 244 nm pump light to excite higher vibrational states in S_1_.

**Figure 6:**
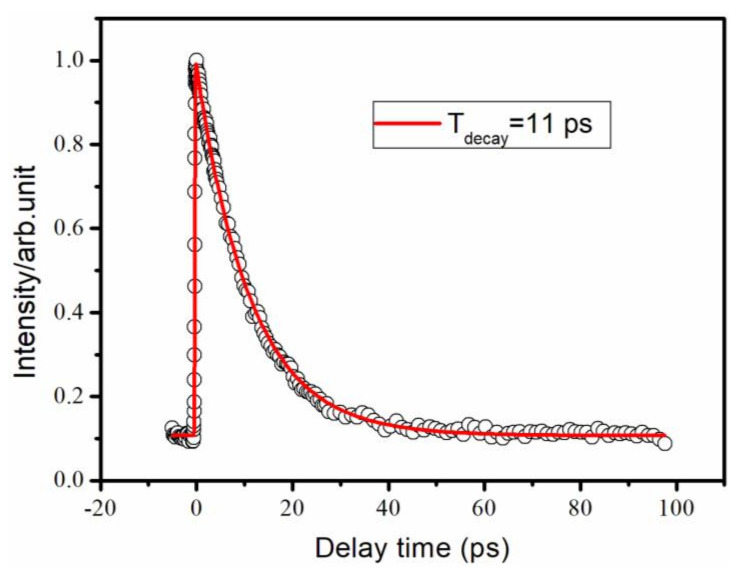
Time decay of the parent ion obtained with 244 nm pump and 400 nm probe detection. The circles are experimental results, and the solid lines are fits with a Gaussian cross-correlation function and a convolution of three exponential decay functions, resulting in different time constants.

Figure 6 shows a signal from the 244 nm pump and 400 nm probe pulses fitted with a convolution of an exponential decay function and a Gaussian function, resulting in a decay time of 11 ps. When the pump light is 244 nm, we did not observe intersystem crossing because the decay of the S_1_ state should have been an internal conversion process directly to the S_0_ state.

As the wavelength gets shorter, the intersystem crossing process probably compete with the internal conversion process. When the pump light was 260 nm, we observed intersystem crossing from S_1_ to T_1_. Although we did not observe internal conversion from S_1_ to S_0_, it most likely occurred. The proportion of the intersystem crossing process appears to be smaller for the 260 nm pump than for the 323 nm pump [31]. However, when the pump light is 244 nm, we did not observe intersystem crossing because the decay of the S_1_ state should have been an internal conversion process directly to the S_0_ state. Therefore, the high vibrational dynamics of the S_1_ state had different decay processes for 260 nm and 244 nm pumping.

**Figure 7:**
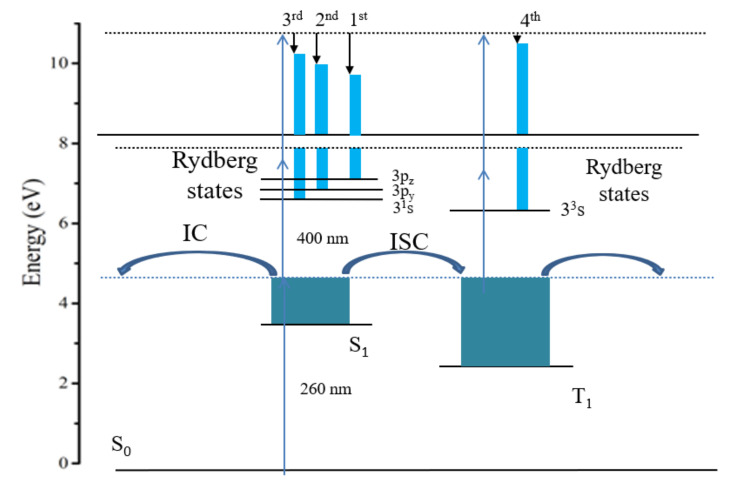
Schematic representation of the excitation and ionization scheme of 2-methlypyrazine using 260 nm pump and 400 nm probe pulses.

The kinetic process of the S_1_ state of the 2-methylpyrazine molecule is summarized in Figure 7. The high vibrational state of the S_1_ state of the 2-methylpyrazine molecule is excited by the 260 nm pump light. When pumping at 260 nm, the first, second, and third peaks come from the ionization of the S_1_ state, and the fourth peak comes from the ionization of the T_1_ state. During the ionization process, the S_1_ and T_1_ states accidentally resonate with the Rydberg state. After the S_1_ state is laid out, the attenuation channel of the S_1_ state is the S_1_→T_1_ intersystem crossing and the S_1_→S_0_ internal conversion process.

## 4. Conclusions

We used a combination of femtosecond time-resolved photoelectron imaging and femtosecond time-resolved mass spectrometry to study the ultrafast radiation-free kinetics of electronic excited states in 2-methylpyrazine. We used 260 nm and 244 nm pump pulses to excite the 2-methylpyrazine to the high vibrational dynamics of the S_1_ state, and we used a 400 nm probe pulse to probe the evolution of that state. By combining both time-resolved mass spectrometry and photoelectron spectroscopy, intersystem crossing from S_1_ to T_1_ was observed, and the layout of the T_1_ state was observed as the S_1_ state decayed. The crossover time scale was 23 ps. At the 244 nm pump wavelength, the S_1_ decay channels laid out by internal conversion competed with each other. With 260 nm pumping, intersystem crossing was still the main decay channel of the S_1_ state; however, with 244 nm pumping, no intersystem crossing was observed, and internal conversion to the ground state was the main decay channel. The channel-three effect occurred during 260 nm excitation because S_1_ internal conversion to the ground state and intersystem crossing to the triplet state competed with each other. The channel-three effect corresponding to 244 nm excitation was only internal conversion from the S_1_ state to the ground state. We also identified the Rydberg states and demonstrated that the photoelectron spectral and angular distributions reflected the accidental resonances of the S_1_ and T_1_ states with the 3s and 3p Rydberg states, respectively, during ionization.

## Figures and Tables

**Table 1:** Energies of electronically excited singlet states and corresponding oscillator strengths calculated by time-dependent density functional theory with a B3PW91/6-311++G (d, p) basis set.

State	Transition	Eexc/eV (cal)	f	Eexc/eV (exp)
S_1_	25→26	3.93	0.0055	3.838
S_2_	25→27	4.69	0.0001	-
S_3_	24→26	5.51	0.1157	-
S_4_	23→26	5.54	0.0000	-
S_5_	25→28	6.25	0.0071	-
S_6_	22→26	6.31	0.0997	-

**Table 2:** Assignments of Rydberg states for 2-methylpyrazine, pyrazine and 2-Picoline.

2-Methylpyrazine		Pyrazine [29]	2-Picoline [30]
Assignment	δ	δ	δ
3py	0.48		0.48
3pz	0.61	0.64	0.62
3^1^s	0.83	0.87	
3^3^s	0.87	0.89	

## Data Availability

Not applicable.

## Sample Availability

Samples of the compounds are not available from the authors.
